# Integrated provision of topical pre‐exposure prophylaxis in routine family planning services in South Africa: a non‐inferiority randomized controlled trial

**DOI:** 10.1002/jia2.25381

**Published:** 2019-09-11

**Authors:** Leila E Mansoor, Nonhlanhla Yende‐Zuma, Cheryl Baxter, Kathryn T Mngadi, Halima Dawood, Tanuja N Gengiah, Natasha Samsunder, Jill L Schwartz, Gustavo F Doncel, Quarraisha Abdool Karim

**Affiliations:** ^1^ Centre for the AIDS Programme of Research in South Africa (CAPRISA) Durban South Africa; ^2^ Eastern Virginia Medical School CONRAD Arlington VA USA; ^3^ Department of Epidemiology Mailman School of Public Health Columbia University New York NY USA

**Keywords:** HIV prevention, pre‐exposure prophylaxis (PrEP), tenofovir, adherence, integration into family planning services, implementation of PrEP scale‐up

## Abstract

**Introduction:**

Tenofovir‐containing oral pre‐exposure prophylaxis (PrEP) is recommended for those at substantial risk as part of combination HIV prevention. However, there are limited data, beyond clinical trial settings, to guide the introduction of PrEP in healthcare services with adequate levels of adherence. Since young women in Africa are at high risk of HIV and likely to utilize family planning (FP) services, the feasibility, acceptability and effectiveness of integrating topical PrEP provision into routine FP services was assessed.

**Methods:**

This two‐arm, randomized controlled, non‐inferiority, open‐label extension trial was undertaken in urban and rural KwaZulu‐Natal, South Africa. HIV‐negative eligible women (n = 372) from the parent trial (Centre for the AIDS Programme of Research in South Africa (CAPRISA) 004) were randomized to receive tenofovir gel either through intervention (FP clinics, n = 189) or control clinics (CAPRISA research clinics, n = 183). Non‐inferiority was predefined as gel use in the intervention clinics would be no more than 20% lower than in the control clinics. Adherence, retention and HIV incidence rates were assessed.

**Results:**

Women were enrolled between November 2012 and October 2014, and followed up for 682.3 women‐years (mean = 22 months). Baseline characteristics of women in intervention and control clinics were comparable and retention rates were 92.1% and 92.3% respectively. Women in intervention clinics and control clinics returned on average 5.2 (95% confidence interval (CI): 4.7 to 5.7) and 5.7 (CI: 5.2 to 6.2) used gel applicators per month respectively, with a mean difference of −0.47 (CI: −1.16 to 0.21). Per‐protocol estimates were on average 5.5 (CI: 5.0 to 6.1) and 5.8 (CI: 5.3 to 6.3) respectively, with a mean difference of −0.25 (CI: −0.98 to 0.48), meeting the non‐inferiority criteria. Adherence, based on proportion of reported sex acts covered by two gel doses, was 79.9% (CI: 76.7 to 83.2) in intervention compared with 73.9% (CI: 70.7 to 77.1) in control clinics; mean difference:6.0% (CI: 1.5 to 10.6) (*p* = 0.009). HIV incidence rates were 3.5 (CI: 1.8 to 6.0) and 3.6 (CI: 1.9 to 6.3) per 100 women‐years in intervention and control clinics respectively. Both these incidence rates were lower than the age‐standardized rate of 6.2 per 100 women‐years (n = 444) in the placebo arm of the parent trial (*p* = 0.019).

**Conclusions:**

Provision of topical PrEP as part of an integrated FP service achieved higher adherence, and was as feasible, acceptable and effective in preventing HIV as provision through a research setting. This provides useful evidence for scale‐up of oral PrEP in urban and rural high burden communities.

## 1 Introduction

The Centre for the AIDS Programme of Research in South Africa (CAPRISA) 004 trial 1 was the first to demonstrate that antiretroviral drugs used vaginally before and after sex could reduce sexually transmitted HIV infection. It also immediately raised the ethical obligations of post‐trial access for trial participants. This trial, and several studies on antiretroviral‐based microbicides (topical product applied inside the vagina or rectum as pre‐exposure prophylaxis (PrEP)) 2, 3, 4, treatment‐as‐prevention 5, and oral PrEP 6, 7, 8, 9, 10, 11 have brought new hope and optimism for HIV prevention efforts, the possibility of epidemic control and an AIDS‐free generation. These series of breakthroughs in HIV prevention, combined with the approval of the first oral antiretroviral drug combination (tenofovir disoproxil fumarate + emtricitabine) for reducing the risk of sexually acquired HIV infection, has led to PrEP being integrated into comprehensive HIV prevention packages in several countries. The best strategy to scale‐up this promising prevention option to those who would benefit the most, without compromising adherence and effectiveness is unknown. Adherence to daily dosing regimens of currently available PrEP, particularly among women, is a significant challenge 12, 13.

In Southern and Eastern Africa, young women are particularly vulnerable to acquiring HIV infection 14. Their vulnerability arises from a complex interplay of biological 15, 16 and social‐behavioural factors 17, that is exacerbated by limited availability of women‐initiated prevention options to reduce their HIV risk 18. PrEP is therefore an important HIV prevention option for this group. Integrating PrEP into existing sexual and reproductive health (SRH) services, such as family planning (FP), is one potential strategy for programmatic access to PrEP.

A key first step is to strengthen existing SRH services to serve as a strong platform for easy PrEP integration. An ideal SRH clinic offers a comprehensive SRH service that includes: customized counselling and testing with regular HIV risk assessment, HIV and pregnancy testing, expanded fertility control method mix provision; cervical cancer screening, gender‐based violence assessment, tuberculosis and sexually transmitted infection (STI) screening, and appropriate treatment and/or referrals when indicated. Given that public health facilities in South Africa are over‐burdened 19, system strengthening for integrated delivery of PrEP will be essential.

The purpose of the CAPRISA 008 trial was to assess the feasibility, acceptability and effectiveness of integrating topical PrEP provision into FP services as one potential strategy to introduce PrEP in the public health sector. We hypothesized that the difference between the mean number of returned used applicators, as a measure of product use, in FP services would be no more than 20% lower than that in a clinical trial setting.

## 2 Methods

### 2.1 Trial design

The CAPRISA 008 trial, a two‐arm, open‐label, randomized controlled, non‐inferiority trial, was conducted between 2012 and 2015 at the urban and rural CAPRISA trial clinics and their neighbouring FP service clinics located in KwaZulu‐Natal, South Africa. The trial design, including eligibility criteria, has been described in great detail elsewhere 20. All participants provided written informed consent.

### 2.2 Randomization and masking

All eligible participants were enrolled within 30 days of screening. At each of the two sites (urban and rural), participants were randomly assigned in a 1:1 ratio to receive tenofovir gel through either FP service clinics (intervention) or CAPRISA trial clinics (control) using permutated blocked randomization of sizes six and eight, stratified by site.

A randomization list, generated by a statistician who was not otherwise involved in the trial, was used to assign individual trial participants to intervention or control clinics. Trial sites were given sealed, opaque randomization envelopes, sequentially labelled with a participant identification number. These envelopes were assigned sequentially to eligible trial participants by the Principal Investigator or designee. Clinic allocation was concealed until after a participant was deemed eligible. As this was an open‐label trial, there was no blinding.

### 2.3 Trial procedures

Provision of tenofovir gel and trial monitoring for women enrolled in the intervention clinics was done through local FP services. Quality improvement (QI) methodology (Model for Improvement and Plan, Do, Study, Act cycle), which utilizes small‐scale rapid cycles of improvement informed by goal setting by local service providers to improve the quality of service delivery 21, 22, 23, was utilized to strengthen the FP services, prior to initiation of CAPRISA 008, to promote reliable delivery of tenofovir gel. An experienced QI advisor coached, mentored and trained FP staff and CAPRISA leadership during the pre‐trial stage by: holding multiple QI workshops which covered topics including principles of QI, problem solving strategies and using local data to make improvements. The QI advisor together with FP staff conducted a gap analysis of existing FP service provision and generated change ideas to improve the quality of FP service delivery in the areas of FP counselling and provision, STI/HIV counselling and treatment, and general clinic processes (e.g. clinic flow, documentation, follow‐up, etc.).

Once FP services were sufficiently optimized, a site initiation assessment was undertaken to ensure readiness for CAPRISA 008 trial initiation and trial participants were subsequently enrolled. All intervention and trial clinic staff were given CAPRISA 008 trial‐specific training. CAPRISA staff provided daily monitoring/trouble shooting for FP clinic staff as they integrated tenofovir gel provision into the FP programmes.

Women in intervention clinics had monthly visits for the first three months post‐enrolment, thereafter gel provision and monitoring was scheduled to coincide with each woman's routine FP visit. Women received gel applicators every two months if utilizing an injectable hormone depot preparation administered two monthly (norethisterone enanthate) or every three months if utilizing an injectable administered three monthly (depot medroxyprogesterone acetate) or any other form of contraception. Women assigned to control clinics received gel applicators monthly, irrespective of frequency or formulation of contraception. Study visits and procedures at control clinics were similar to those followed in the CAPRISA 004 trial 1. A summary of the similarities and differences between the intervention and control clinics is provided in Table 1. Self‐reported product and service acceptability assessments were conducted six‐monthly in all clinics.

**Table 1:** Similarities and differences between the intervention and control clinics

Similarities
CAPRISA 008 trial specific training on the protocol, study specific procedures manual, standard operation procedures and completion of case report forms. A comprehensive prevention package comprising education, adherence counselling, condom promotion, STI treatment and HIV testing was provided. FP and reproductive health services were provided Tenofovir gel was provided Intensive six‐monthly monitoring visits were conducted Safety monitoring at every study visit was conducted

Differences	
Intervention clinic	Control clinic
Attended the QI‐strengthened FP services throughout the trial Received all services, including tenofovir gel provision, from FP clinic staff, under the guidance of a quality mentor Clinic visits were three monthly, except for women on NET‐EN where visits were two monthly Tenofovir gel provision was integrated into FP service provision	Attended CAPRISA clinics throughout the trial Received all services, including tenofovir gel provision, from CAPRISA clinic staff Clinic visits were monthly Tenofovir gel was provided separately not integrated into FP service provision

CAPRISA, Centre for the AIDS Programme of Research in South Africa; FP, family planning; QI, quality improvement; STI, sexually transmitted infection.

HIV and pregnancy testing was performed at each study visit, using the Determine HIV 1/2 (Abbott Laboratories, Lake Bluff, IL, USA) and Uni‐Gold Recombigen^®^ (Trinity Biotech, Wicklow, Ireland) HIV rapid tests and the QuickVue One‐Step hCG Urine pregnancy test (Quidel Corporation, San Diego, CA, USA). HIV/STI risk reduction messages, condoms, contraceptive services and counselling on product adherence were provided to all participants. In addition to the one‐on‐one motivational interviewing adherence support sessions 24, the CAPRISA 008 trial incorporated group adherence support sessions. Trial participants were counselled and supported throughout the trial to adhere to a pre‐ and post‐coital dosing strategy, also referred to as BAT 24 1, 25, that is apply the first dose of the assigned study gel within 12 hours before anticipated sex; a second dose as soon as possible but within 12 hours after sex and to apply no more than two gel doses in 24 hours. Participants in both trial arms were requested to return all used and unused applicators at each study visit.

While contraceptive services were provided to all CAPRISA 008 participants, those who became pregnant during the trial discontinued product use, but were advised to continue with their follow‐up visits. When these participants no longer had a positive pregnancy test, the pregnancy outcome was documented and they were re‐started on tenofovir gel if they wished to continue with trial participation.

Clinical safety was assessed at baseline and throughout the trial. Participants infected with the hepatitis B virus at enrolment were closely monitored using laboratory diagnostics, especially during episodes of product hold (i.e. when trial product is withheld from a participant who is still enrolled in the trial). Pelvic examinations, including collection of blood and genital specimens, were conducted at enrolment, six monthly, study exit and when clinically indicated to assess secondary outcomes, including product adherence. Tenofovir concentrations in the genital tract were measured at 12 months in 313 vaginal aspirate samples (n = 157 in intervention clinics and n = 156 in control clinics) using a validated ultra‐performance liquid chromatograph mass spectrometry method 26. Sample processing and analysis, including tenofovir threshold concentrations, were done according to methods previously described 27. For any adverse symptoms experienced between scheduled visits, participants were counselled to report to their assigned clinic as soon as possible. Any participant needing further treatment was referred to a healthcare provider for follow‐up care.

Participants identified with an STI or other treatable reproductive tract infection were provided counselling and clinical care at their assigned clinics in accordance with the South African Department of Health guidelines. Participants with STIs were encouraged to refer their partners for treatment.

Participants who acquired HIV infection during trial follow‐up were referred to the CAPRISA Acute Infection Study (CAPRISA 002) or CAPRISA Treatment Study (CAPRISA 009) for ongoing care, antiretroviral therapy and follow‐up. Participants who did not wish to enroll in these CAPRISA studies were referred to appropriate health facilities, serving the catchment populations, for required care and support.

### 2.4 Statistical analysis

Based on data from CAPRISA 004 1, the mean number of returned used applicators per participant per month was anticipated to be 5 (SD = 3). The primary objective of this non‐inferiority trial was to demonstrate that gel use (measured by mean number of returned used applicators per participant per month) in intervention clinics was not more than 20% lower (i.e. difference is not more than one applicator per participant per month) than control clinics, thereby supporting feasibility of integrating PrEP provision in intervention clinics. Given that this trial was an open‐label extension of the CAPRISA 004 trial, a maximum of 700 women (350 in each arm) could be enrolled. This sample size provided >90% power to demonstrate whether gel use in women in intervention clinics was similar to, but not more than 20% lower than gel use by women in control clinics, adjusted for 10% loss to follow‐up. That is, the lower bound of the 95% CI does not exceed 20% of the mean returned used applicators in control clinics.

The primary endpoint was compared using linear mixed model with compound symmetry structure. Non‐inferiority was tested by intention‐to‐treat (included 5759 study visits) and per‐protocol analyses (included 5289 study visits) using the 95% CI of the difference in mean returned used applicators. The per‐protocol analysis excluded visits where no gel had been dispensed for >120 days. Fisher's exact test was used for the analysis of categorical data, and unpaired t‐tests or the Wilcoxon two‐sample test for analysis of continuous data. We used Poisson approximations to calculate 95% CIs for incidence rates and the F test to calculate 95% CIs for incidence rate ratios (IRR). All data analysis were undertaken using SAS version 9.4 (SAS Institute Inc., Cary, NC, USA).

This trial was conducted under regulatory oversight of the South African Medicines Control Council (20110145), ethical oversight of University of KwaZulu‐Natal's Biomedical Research Ethics Committee (BFC 237/010) and Chesapeake Institutional Review Board (Pro00007432), and registered with the South African Department of Health (DOH‐27‐0812‐4129) and ClinicalTrials.gov (NCT01691768).

## 3 Results

### 3.1 Trial population

**Figure 1:**
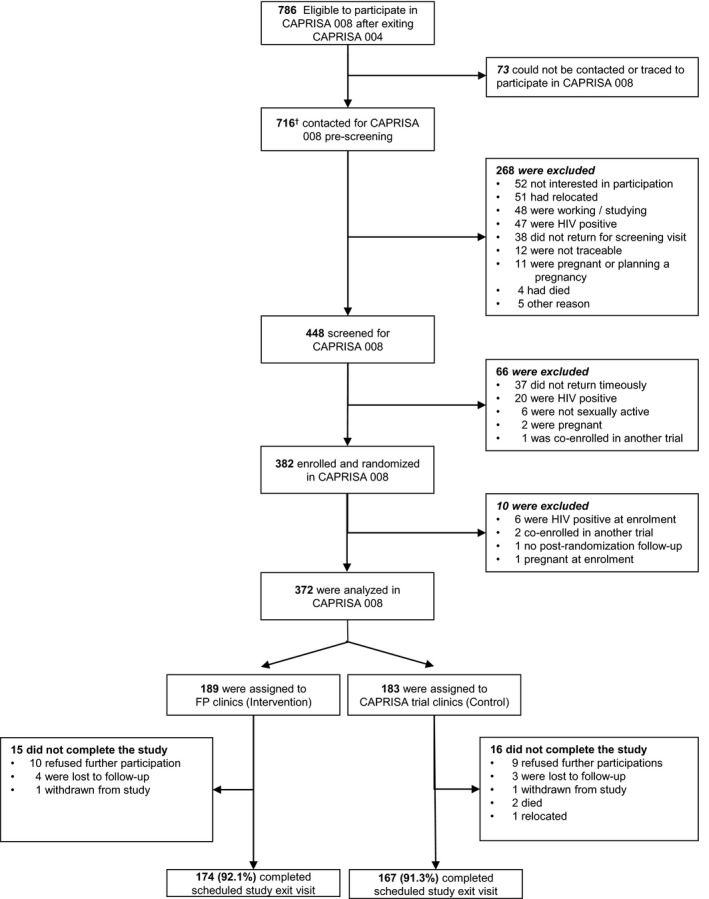
Screening, enrolment, randomization and follow‐up of the trial participants ^†^This includes three participants who were screened for CAPRISA 008 in error; one was screened out and two were enrolled. CAPRISA, Centre for the AIDS Programme of Research in South Africa.

Of the 889 women enrolled in CAPRISA 004, 786 women were known HIV‐negative participants at the end of the trial and subsequently eligible for participation in CAPRISA 008 (Figure 1). Of these, 716 were contactable and invited to screen. Of these, 448 screened for participation and 382 (266 rural and 116 urban) women were enrolled and randomized, of whom 372 women were included in the analysis. A total of 189 women were randomized to intervention clinics – 27 women followed the two monthly schedule and 162 women followed the three monthly schedule – and 183 women were randomized to control clinics. Women were enrolled between 07 November 2012 and 16 October 2014, and followed up for 682.3 women‐years (mean = 22 months). Retention rates, measured over the maximum follow‐up period of 28 months, were similar between intervention (92.1% (174/189)) and control clinics (92.3% (169/183)), and remained above the target of 90% per annum.

At enrolment, the demographic characteristics and sexual behaviour of women in intervention clinics (n = 189) and control clinics (n = 183) were similar (Table 2). One hundred and twenty eight women were initiated on a non‐barrier method of contraception at baseline; 61 (32.3%) in intervention clinics and 67 (36.6%) in control clinics. HSV‐2 prevalence was high overall (>88%) and was higher in intervention than control clinics. The mean age of women enrolled in CAPRISA 008 was 29.4 ± 5.6 (range: 20‐44), at least half of the women (n = 216 (58.1%)) had completed high school and most women (n = 278 (74.7%)) were using a hormonal injectable contraceptive.

**Table 2:** Baseline characteristics of enrolled participants

Variable	Variable category	Intervention clinics (N = 189)	Control clinics (N = 183)	*p*‐Value
Socio‐demographic characteristic				
Age (mean; ±SD; range)		29.5; ±5.8; 20‐44	29.3; ±5.3; 22‐44	0.741
Parity (mean; ±SD; range)		1.6; ±1.1; 0‐8	1.5; ±1.0; 0‐5	0.451
Education level (n, %)	Did not complete high school	78 (41.3)	78 (42.6)	0.973
	High school completed	98 (51.9)	92 (50.3)	
	Tertiary education initiated	13 (6.9)	13 (7.1)	
Initiated on contraception (n, %)		61 (32.3%)	67 (36.6%)	0.385
Contraception (n, %)	Depot medroxyprogesterone acetate	111 (58.7)	111 (60.7)	0.223
	Hormonal oral	43 (22.8)	32 (17.5)	
	Norethisterone enanthate (NET‐EN)	27 (14.3)	29 (15.8)	
	Implant	1 (0.5)	0 (0.0)	
	Tubal ligation	5 (2.6)	11 (6.0)	
	Hysterectomy	2 (1.1)	0 (0.0)	
Sexual behavioural characteristics				
Total lifetime sex partners (mean; ±SD; range)		3; ±2.5; 1‐25	3; ±2.7; 1‐20	0.745
Sex acts in the past 30 days (mean; ±SD; range)		6; ±6.7; 0‐72	6; ±5.9; 0‐38	0.970
Anal sex in the past 30 days (n/N, %)		1/188(0.5)	2/183 (1.1)	0.619
Living with regular partner (n/N, %)		35/187 (18.7)	38/183 (20.8)	0.695
New sex partner in the last 30 days (n, %)		0	0	0.619
Partner of last 30 days tested positive for HIV (n, %)		6 (3.2)	3 (1.6)	0.367
Male condoms use (n, %)	Always	65 (34.4)	78 (42.6)	0.163
	Sometimes	95 (50.3)	86 (47.0)	
	Never	29 (15.3)	19 (10.4)	
Clinical characteristics				
Genital symptoms in the last 30 days (n, %)		49 (25.9)	42 (23.0)	0.547
HSV‐2 prevalence (n/N, %)		174/182 (95.6)	159/179 (88.8)	0.018
HPV prevalence (n/N, %)		20/186 (10.8)	11/179 (6.1)	0.134

HPV, human papillomavirus; HSV‐2, Herpes simplex viruse type 2.

### 3.2 Tenofovir gel use, adherence and drug levels by trial arm

Overall 84,260 gel applicators were dispensed and 83,052 (98.6%) were returned to the clinics; 37,250 (44.9%) in intervention clinics and 45,802 (55.1%) in control clinics. Of these, 42,650 (50.6%) applicators were returned as used (19,631 (51.6%) in intervention clinics and 23,019 (49.8%) in control clinics) and 40,402 (47.9%) applicators were returned as unused (17,619 (46.3%) in intervention clinics and 22,783 (49.3%) in control clinics).

Overall, women returned an average of 5.5 used applicators and reported a mean of 4.1 sex acts monthly. In the intention‐to‐treat analysis, the mean monthly returned used applicators were 5.2 (CI: 4.7 to 5.7) in intervention clinics compared with 5.7 (CI: 5.2 to 6.2) in control clinics, with a mean difference of −0.47 (CI: −1.16 to 0.21) (Table 3). Per‐protocol estimates were 5.5 (CI: 5.0 to 6.1) and 5.8 (CI: 5.3 to 6.3) in intervention and control clinics respectively, with a mean difference of −0.25 (CI: −0.98 to 0.48). In the intention‐to‐treat analysis, non‐inferiority (lower than 20% use or one applicator) was inconclusive; however, in the per‐protocol analysis non‐inferiority was achieved.

**Table 3:** Adherence (gel/sex ×2), drug levels and HIV incidence of enrolled participants

	Intervention clinics (N = 189) (95% CI)	Control clinics (N = 183) (95% CI)
Adherence		
Intention‐to‐treat: mean returned used applicators per month	5.2 (4.7 to 5.7)	5.7 (5.2 to 6.2)
Mean difference	−0.47 (−1.16 to 0.21)	
Per‐protocol: mean returned used applicators per month	5.5 (5.0 to 6.1)	5.8 (5.3 to 6.3)
Mean difference	−0.25 (−0.98 to 0.48)	
Mean number of sex acts per month	3.6 (3.2 to 4.1)	4.5 (4.0 to 5.0)
Mean difference	−0.90 (−1.46 to −0.16)	
Mean adherence^a^	79.9% (76.2 to 83.2)	73.9% (70.7 to 77.1)
Mean difference	6.0 (1.5 to 10.6)	
Drug levels		
Proportion with detectable drug levels at 12 months of follow‐up	39.5% (32.2 to 47.3)	43.6% (36.1 to 51.4)
Risk ratio	0.91 (0.70 to 1.18)	
% with detectable drug levels when sex is reported within seven days	36/70 (51.4%)	45/69 (65.2%)
	81/139 (58.3%)^b^	
% with detectable drug levels when no recent sex is reported	26/87 (29.9%)	23/87 (26.4%)
	49/174 (28.2%)^b^	
HIV incidence		
HIV incidence per 100 women‐years	3.5 (1.8 to 6.0)	3.6 (1.9 to 6.3)
Incidence rate ratio	0.96 (0.40 to 2.35)	

a Estimated proportion of reported sex acts covered by two gel doses

b Represents overall proportion for the two arms combined.

The mean monthly reported sex acts were 3.6 (CI: 3.2 to 4.1) in intervention clinics compared with 4.5 (CI: 4.0 to 5.0) in control clinics (Table 3), with a mean difference of −0.90 (CI: −1.46 to −0.16) (*p* = 0.014). Mean adherence, defined as the estimated proportion of reported sex acts covered by two gel doses 1, 25, was significantly higher in intervention compared with control clinics (79.9% (CI: 76.2 to 83.2) versus 73.9% (CI: 70.7 to 77.1), mean difference = 6.0%) (*p* = 0.009). Most of the participants in both intervention (70.2%) and control clinics (65.2%) were able to use two doses of gel for more than 80% of their sex acts.

Tenofovir was detectable in genital fluid at month 12 in 62 of 157 women (39.5%) at intervention clinics and 68 of 156 women (43.6%) at control clinics. However, genital tenofovir was detected in 81 women (58.3%) of the 139 women who reported sex in the week prior to the clinic visit (51.4% in intervention clinics and 65.2% in control clinics; *p* = 0.122) and in 49 women (28.2%) of the remaining 174 women who reported no recent sex (29.9% in intervention clinics and 26.4% in control clinics; *p* = 0.736) (Table 3).

### 3.3 HIV incidence

During the trial there were 24 seroconversions; 12 in control clinics and 12 in intervention clinics. The HIV incidence rates were 3.5 per 100 women‐years in intervention clinics and 3.6 per 100 women‐years in control clinics (IRR: 0.96, CI: 0.40 to 2.35, *p* = 0.928) (Table 3). HIV incidence rates in women who had detectable tenofovir drug levels (n = 130) at 12 months was 0.8 per 100 women‐years (CI: 0.1 to 2.8) and in women whose drug levels were below the level of quantification (BLQ) (n = 183) was 3.1 per 100 women‐years (CI: 1.6 to 5.6). There were two seroconversions among those with detectable drug levels and 11 among those with levels BLQ (*p* = 0.069).

### 3.4 Product and service acceptability

Of the 372 women analysed, 360 had complete data on product and service acceptability. Gel acceptability among these women was high (>95%), with the top five reasons for using the gel being: HIV protective benefits (n = 269 (74.7%)), perceived vaginal cleansing properties (n = 121 (33.6%)), ease of use (n = 94 (26.1%)), enhanced sexual pleasure (n = 80 (22.2%)), and perceived protection against STIs (n = 70 (19.4%)). While the majority of women (n = 262 (72.8%)) expressed acceptability of the product, a few indicated the following negative properties of the gel: messy (n = 41 (11.4%)), too much lubrication (n = 20 (5.6%)) and product leakage (n = 27 (7.5%)). The majority of women (n = 310 (86.1%)) reported disclosing their trial participation to at least one sexual partner and of those, 97.1% (n = 301) had disclosed product use to their partner.

At study exit, 80.3% (n = 147/183) of women in intervention clinics and 75.1% (n = 133/177) in control clinics indicated that they were happy receiving HIV preventative care together with their fertility control services from a FP facility (Table 4).

**Table 4:** Participant preference for receiving HIV prevention^a^

	Total (N = 360) % (N)	Intervention clinics (N = 183) % (N)	Control clinics (N = 177) % (N)
Private general practitioner	7.5 (27)	6.0(11)	9.0 (16)
Public sector primary healthcare/family planning clinics	77.8 (280)	80.3 (147)	75.1 (133)
Mobile clinics	1.7 (6)	2.7 (5)	0.6 (1)
Does not want to use trial product	0.3 (1)	0.0 (0)	0.6 (1)
Other^b^	12.8 (46)	10.9 (20)	14.7 (26)

CAPRISA.

a 12 women had missing data

b Other = CAPRISA clinics (44), pharmacy (1), a place that opens on weekends (1).

## 4 Discussion

In this trial, we showed that mean returned used applicators in intervention clinics was not more than 20% lower than in control clinics. Although the non‐inferiority criteria was not met in the intention‐to‐treat analysis, the per‐protocol analysis, which excluded visits where no gel was dispensed for >120 days, met the non‐inferiority criteria demonstrating that accessing topical PrEP from FP clinics was non‐inferior to a clinical trial setting in terms of gel use. Adherence has been shown to be a major challenge in several topical 2, 3, 4 and oral PrEP efficacy trials 6, 7, 8, 9, 10, 11 in women. Given that the primary outcome measure is adherence, the per‐protocol analysis may be a better reflection of actual gel use.

The dosing regimen used in this trial was peri‐coital, therefore a measure of adherence that factored in the proportion of reported sex acts covered by two gel doses also needed to be considered. Using this adherence measure, adherence was significantly higher (mean difference = 6.0%) in the intervention than control clinics. Based on genital tenofovir concentrations, adherence was marginally lower in intervention compared with control clinics. The slightly lower drug levels in intervention clinics were not surprising, since women attending FP services reported less frequent sex and returned fewer used applicators compared with women in trial clinics. In addition, more than 75% of women expressed a preference for receiving HIV prevention from intervention clinics. Taken together, these data suggest that integration of PrEP into existing public healthcare facilities is feasible and does not compromise adherence.

The overall HIV incidence rate of 3.5 per 100 women‐years observed in the CAPRISA 008 trial was considerably lower than the incidence rate of 9.1 per 100 women‐years in the placebo arm and 5.6 per 100 women‐years in the tenofovir gel arm (IRR = 0.61; *p* = 0.017) of the CAPRISA 004 trial 1. However, the women in CAPRISA 004 and CAPRISA 008 may not be directly comparable as their risk profiles may have changed over time. The absence of a contemporaneous placebo‐control group is an important limitation of this trial. As a placebo‐control group would be unethical in a post‐trial access study, rigorous approaches to estimate what HIV incidence would have been in the absence of the intervention need to be applied. Such approaches have been previously applied in other open‐label PrEP studies 4, 28. For this trial, the placebo‐control group of the CAPRISA 004 trial, which was undertaken in the same communities a few years earlier, served as the external comparison group. Using an age‐standardized analysis, the HIV incidence rate in the placebo group (n = 444) was 6.2 per 100 women‐years. Thus, the overall HIV incidence rate was 44% lower than that observed in an age‐comparable historical placebo‐control group. In an adhoc analysis of the cohort comparing women with detectable versus non‐detectable tenofovir in genital fluid, very few infections occurred in the former group showing consistent use of tenofovir gel (Incidence rate = 0.8 vs. 3.1 per 100 women‐years respectively).

The trial was designed on a sample size of 700 women, however, only 372 women were eligible for study participation. This study limitation was a consequence of several years having elapsed between the end of CAPRISA 004 and the late start of CAPRISA 008 due to lengthy delays in regulatory approvals. Regulatory authorities now require a post‐trial access plan to be included in the original trial application. This will avoid delays in initiating the post‐trial open‐label extension and allow participants rapid access to the efficacious intervention. During the time elapsed between the end of CAPRISA 004 and the start of CAPRISA 008, there were 73 new HIV infections (among CAPRISA 004 trial participants who were contactable) with an estimated incidence of 34%.

Furthermore, this trial utilized a peri‐coital topical PrEP strategy, which may not be directly applicable to daily oral PrEP implementation. The FACTS 001 trial 29, also assessing a topical PrEP strategy, did not confirm the results of CAPRISA 004. It is therefore unlikely that tenofovir gel will be licensed as an HIV prevention technology. While some of the behaviours related to PrEP may be formulation dependent, this trial provides information that is broadly applicable to the motivation of women to initiate, maintain follow‐up and adhere to PrEP incorporated into FP services. Oral PrEP is now part of the HIV prevention package recommended by the World Health Organization and should be made available to women, especially in high HIV burden countries. Integration into FP services could provide one strategy for reaching women who want to use PrEP. In addition, we recognize that for this trial, women were already educated on how to use a vaginal gel by participating in the parent CAPRISA 004 trial; they did not require additional training by clinic staff in how to use a new product. However, for future rollout of new PrEP products additional time, resources, and training will be an essential factor to consider for future product delivery.

The QI methodology was used as a pre‐trial strategy to strengthen the FP services. This strategy remains a robust and easily transferable approach in the current context of prevention and FP service delivery, and has been successfully applied in resource limited settings (including South Africa) to improve the performance of prevention of mother‐to‐child HIV transmission programmes 30, 31, 32, reducing infant and neonatal mortality 33, 34 and increase coverage of HIV services 35. A similar strategy may provide a locally sustainable approach to integration of PrEP provision. Importantly QI methodology promotes ownership for the quality of service delivery, nurturing critical thinking and problem solving skills, thereby empowering healthcare providers in the face of overwhelming service delivery challenges. Integrating HIV prevention and FP services using a health systems strengthening approach is particularly applicable in a South African setting and has several advantages. These include: large numbers of sexually active women, who would benefit from PrEP provision already utilize FP services at regular intervals, FP staff are knowledgeable about reproductive health and FP staff have experience providing counselling and adherence support. Use of antiretrovirals may have an impact on fertility or pregnancy outcomes, for example dolutegravir 36 and healthcare providers prescribing PrEP should be knowledgeable in both subjects.

Several countries are implementing oral PrEP as part of a comprehensive HIV prevention package 37. In South Africa, PrEP was approved by the local regulatory authority in 2015 38, and is being made available in the public sector to selected high‐risk populations at a small number of pilot public facilities and at some tertiary education institutions. As of 01 May 2019, there are an estimated 13,500 individuals initiated on oral PrEP in South Africa, which is below the country target of 28,099 37. To reach these targets massive scale‐up will be required and strengthened FP services will likely play an essential role.

These data supports the integration of PrEP into existing public healthcare services promoting scale‐up for at‐risk populations, like young women. As with fertility control, women are diverse and will have different PrEP needs at different times in their lives. Efforts currently underway to expand formulations and product delivery forms of PrEP, including long acting, slow release products, which may address adherence challenges, are important to ensure an array of safe and efficacious prevention options for women. Once licensed, integration of these products into FP services may allow for convenient access to much needed HIV prevention modalities for women. In the recent Evidence for Contraceptive Options and HIV Outcomes study 39 HIV risk factors were not part of the enrolment criteria; however, the overall HIV incidence rate was high (3.81%). Participants were sexually active young women looking for contraception. This further emphasizes that HIV prevention services should be integrated into FP services.

## 5 Conclusions

The CAPRISA 008 trial adherence, drug levels, retention and HIV incidence rates were similar between the intervention and control clinics. Among women continuing PrEP use, our trial demonstrated that integrating PrEP into QI strengthened FP services is feasible acceptable, and effective for South African women utilizing these services. This evidence may be of utility to policy makers and healthcare providers implementing PrEP scale‐up.

## Competing interests

QAK is a co‐inventor on pending patents, PCT61/354.050 and PCT 61/357,892 (filed in 2011), for tenofovir gel against HSV‐1 and HSV‐2. JLS and GFD are employed at CONRAD, the regulatory sponsor and manufacturer of tenofovir gel. All other authors have no competing interests.

## Authors’ contributions

LEM contributed to conception and design, trial management, data collection and manuscript writing. NY provided technical advice for statistical aspects and data analysis. CB contributed to conception and design and manuscript writing. KTM and HD contributed to trial coordination and data collection. TNG provided technical advice for pharmacy aspects and data collection. NS provided technical advice for laboratory aspects and data collection. JLS, GFD and QAK contributed to conception, design and critical revision of the manuscript. All authors read and approved the final manuscript.
